# Emergence of mass spectrometry detergents for membrane proteomics

**DOI:** 10.1007/s00216-023-04584-z

**Published:** 2023-02-18

**Authors:** Jan-Simon Behnke, Leonhard H. Urner

**Affiliations:** Department of Chemistry and Chemical Biology, TU Dortmund University, Otto-Hahn-Str. 6, 44227 Dortmund, Germany

**Keywords:** Membrane, Protein, Detergent, Mass spectrometry, Omics

## Abstract

Detergents enable the investigation of membrane proteins by mass spectrometry. Detergent designers aim to improve underlying methodologies and are confronted with the challenge to design detergents with optimal solution and gas-phase properties. Herein, we review literature related to the optimization of detergent chemistry and handling and identify an emerging research direction: the optimization of mass spectrometry detergents for individual applications in mass spectrometry–based membrane proteomics. We provide an overview about qualitative design aspects including their relevance for the optimization of detergents in bottom-up proteomics, top-down proteomics, native mass spectrometry, and Nativeomics. In addition to established design aspects, such as charge, concentration, degradability, detergent removal, and detergent exchange, it becomes apparent that detergent heterogeneity is a promising key driver for innovation. We anticipate that rationalizing the role of detergent structures in membrane proteomics will serve as an enabling step for the analysis of challenging biological systems.

## Introduction

Proteomics aims for the identification and quantification of the proteome (entirety of all proteins) in cells or organisms at a certain time point. On a cellular level, interactions between proteins and other molecules are frequently translated into biological function. Therefore, proteomics is an important tool for describing biological systems and plays a crucial role in disease-relevant research fields, such as the search for new biomarkers, drug discovery, and monitoring disease development [1, 2]. However, various parameters make the analysis of the proteome challenging. The proteome of cells is dynamic and changes in response to its environment [3]. Furthermore, cells are compartmentalized by membranes. Water-insoluble membranes separate water-soluble compartments with different functions. Complementary, the proteome is divided into a water-soluble proteome and a membrane-bound proteome. Both sub-proteomes differ in terms of solubility and abundance. Even though the membrane-bound proteome marks only 30% of the genetically encoded proteome, it accounts for 60% of current drug targets [4, 5]. Technologies that enable the investigation of interactions between drugs and the membrane-bound proteome are of great interest in the pharmaceutical industry [4–7].

**Fig. 1:**
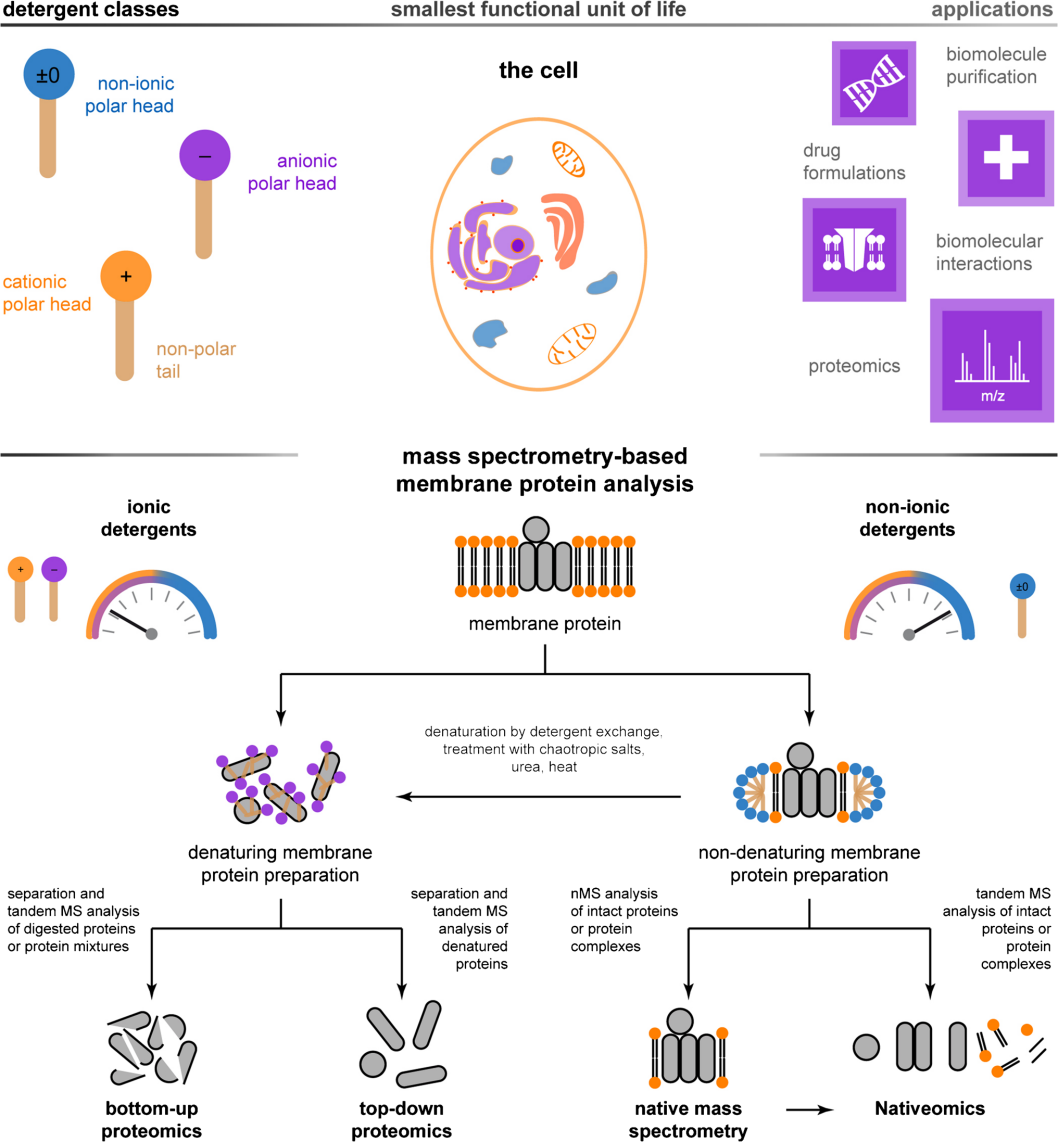
Mass spectrometry detergents for mass spectrometry–based membrane protein analysis. Schematic overview about detergent classes, e.g., non-ionic (blue), cationic (orange), or anionic (purple), a cell, and applications in life sciences that require detergents and aim for a better understanding of how a cell works (upper panel). Furthermore, a schematic overview about possible applications and related MS detergent classes involved in mass spectrometry–based membrane protein analysis is shown (lower panel)

Mass spectrometry(MS)–based proteomics is frequently used to study interactions between the membrane-bound proteome and drugs [8, 9]. MS methods require analytes to be homogenously dissolved. However, this is the opposite to what cells are (Fig. 1). Sample preparation procedures are tailored to overcome problems associated with heterogeneity, low solubility, and abundance of the membrane-bound proteome. Depending on the MS-based proteomics approach, sample preparation procedures include the use of chaotropic salts, organic solvents, organic acids, and detergents [8, 10, 11].

In this review, we focus on detergents related to four established MS-based proteomics approaches: bottom-up proteomics (BUP), top-down proteomics (TDP), native mass spectrometry (nMS), and Nativeomics [12–14] (Fig. 1). In BUP, proteins or protein mixtures are digested by proteases. Peptide fragments are separated by liquid chromatography (LC) and analyzed by tandem MS. Peptide fragment identity is confirmed by matching experimental gas-phase fragmentation patterns with those available from databases or prediction tools [15]. In TDP, proteins or protein mixtures are denatured, subunits are separated by LC, and identified by tandem MS (Fig. 1). BUP and TDP are used to study proteome changes in response to stimuli, which recently enabled the analysis of the rising NQO1 abundance at increasing CTE stages [16], the protective role of Ola1p in yeast cells during heat shock [17], understanding kinase inhibitor mechanisms [18], identification of proteoforms and phosphorylation sites at kinase subunits [19], and age-dependent changes of the human pancreas proteome [20]. These applications demonstrate not only the utilities of BUP and TDP to analyze the proteome of target ecosystems, but also underline their relevance for clinical research and fundamental research in biology more widely.

In contrast to BUP and TDP, nMS-based workflows aim for the preservation of non-covalent interactions in protein assemblies during sample preparation and MS analysis. The idea is to bring protein assemblies, including non-covalently bound ligands, intact into the vacuum of a mass spectrometer, to study structural organization on the level of the whole assembly (Fig. 1). This includes the analysis of protein oligomer equilibria in response to ligand binding [21, 22], gas-phase unfolding stability in response to PTMs or ligand binding [23, 24], relative dissociation constants of protein-lipid binding events [25], cooperativity in protein–ligand binding [25, 26], and the effect of salt microenvironment surrounding membrane proteins on ligand binding [27]. More recently, a multistage nMS method has been developed to identify and characterize lipids, peptides, or therapeutics in direct contact with membrane proteins (Fig. 1) [14]. This method, also known as Nativeomics, uses an Orbitrap Eclipse Tribrid mass spectrometer to dissociate ligands from protein complexes and enables their detailed identification by tandem MS. Conveniently, what has been a combination of two methods, e.g., nMS and omics (lipidomics, proteomics, metabolomics), is now unified in one method, i.e., Nativeomics [14].

Regardless of the MS-based proteomics approach, detergents are a critical enabling step for the analysis of the membrane-bound proteome. Even though detergents enable life sciences in many ways, be it for the purification of biomolecules, drug formulations, or biomolecular interactions studies, the best detergents are selected empirically (Fig. 1) [28]. This dogma also accounts for the selection of detergents in MS-based membrane proteomics [29].

Detergent designers face the challenge to identify design rules that lead to detergents with optimal solubilizing, denaturing, and MS-compatible properties for individual MS-based membrane proteomics applications (Fig. 1). The desire to compromise this set of detergent properties is unique to MS-based membrane proteomics and encouraged us to coin a new category for related detergents in the field, namely mass spectrometry detergents (MS detergents). Herein, we identify an emerging research trend, i.e., the development of tailor-made MS detergents for individual applications in MS-based membrane proteomics. We review recent discoveries in the field to facilitate the predictable optimization of MS detergents for the MS-based analysis of the membrane-bound proteome.

## Methods

A PubChem-based search for English-written literature using the search term “detergents proteomics” [Title/Abstract] OR “surfactants proteomics” [Title/Abstract] AND 2018/01/01 [Date—Publication]: 2022/06/20 [Date—Publication]” was performed on 20 June 2022. Additionally, scientific literature and reference lists of publications within the scope of the current article were mined to identify relevant, but not PubChem-listed publications. Literature related to nMS and Nativeomics has been selected based on the authors’ experience.

## Results and discussion

### Designing detergents for BUP and TDP

**Fig. 2:**
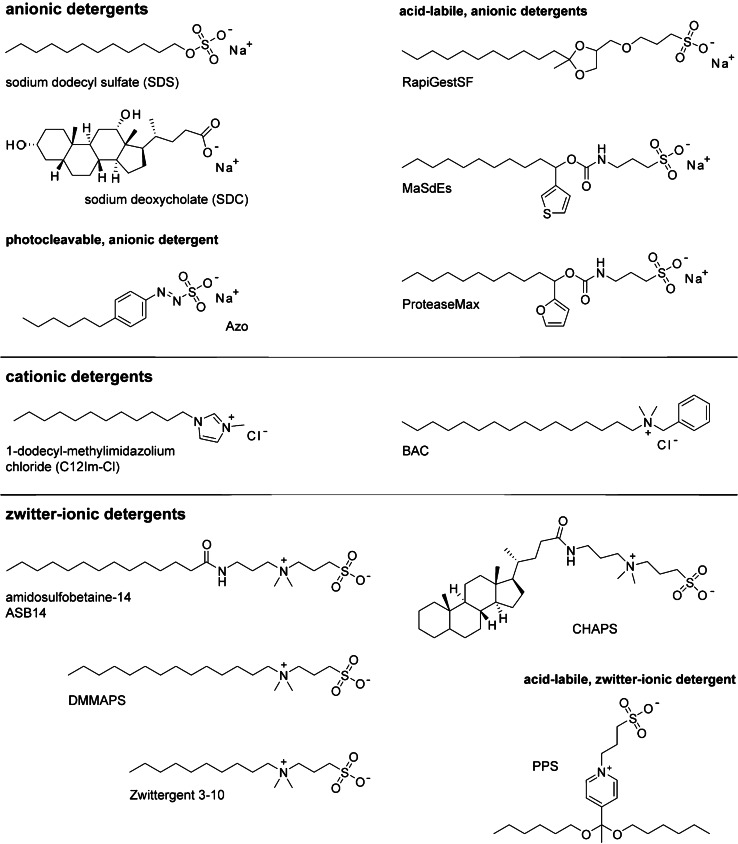
Mass spectrometry detergents for denaturing BUP and TDP experiments. Overview about detergent classes employed for denaturing BUP and TDP experiments, which are mainly anionic, cationic, and zwitter-ionic

**Fig. 3:**
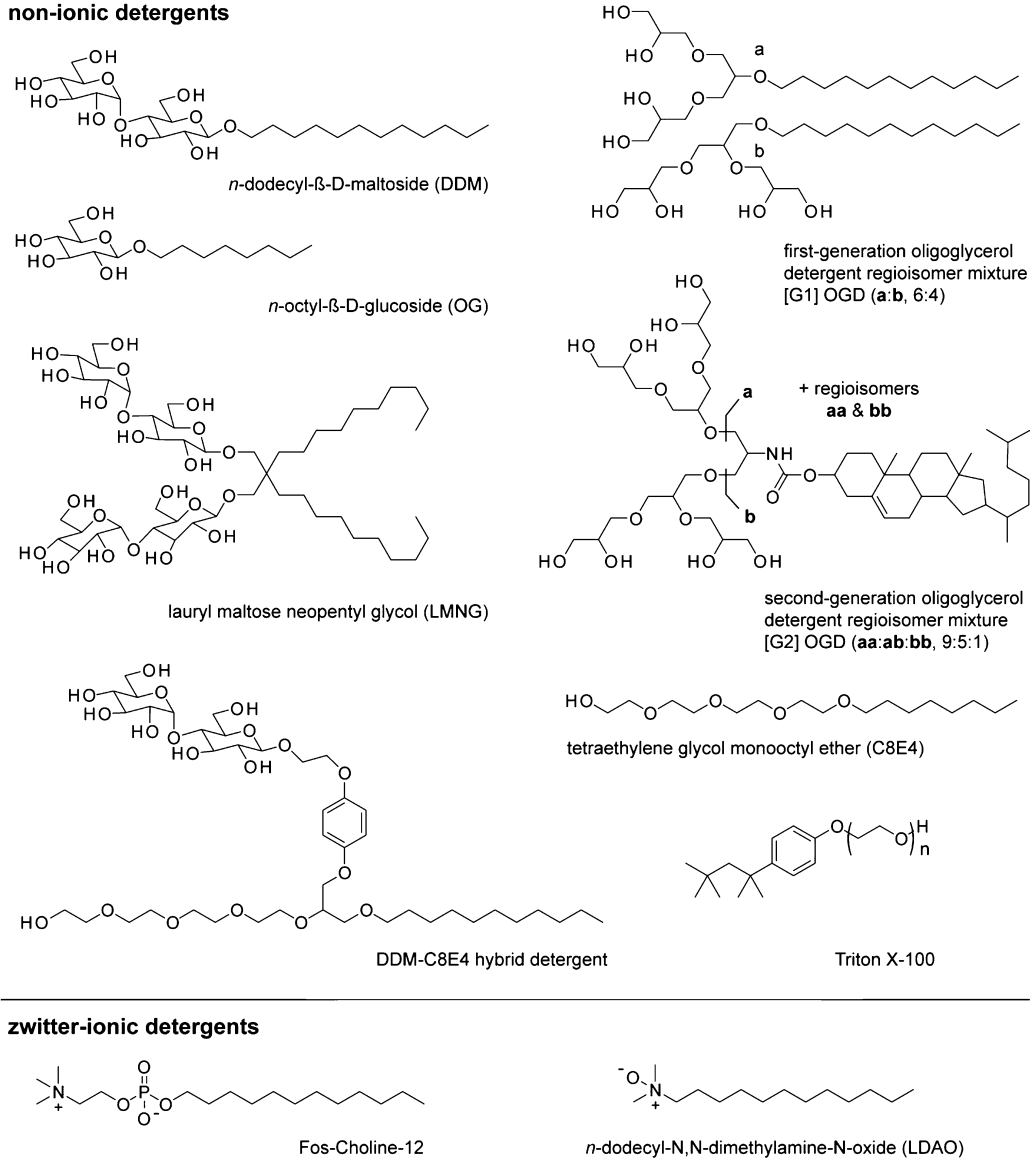
Mass spectrometry detergents for nMS and Nativeomics experiments. Overview about detergent classes employed for non-denaturing nMS and Nativeomics experiments. Related detergent classes are mainly non-ionic or zwitter-ionic

In BUP and TDB experiments, ideally, all proteins are solubilized and isolated from cells in the first step, since unwanted interactions with impurities or insolubilized material could interfere with the MS measurements. While cytosolic proteins are mostly water-soluble, membrane proteins can pose an issue. Their low water solubility generally hinders quantitative solubilization and homogenization by mechanical means. Chaotropic salts, like urea or guanidine, can enhance solubility, but they often do not quantitatively solubilize hydrophobic membrane proteins and bias relative abundances of observable protein populations [8, 10]. Instead, ionic detergents are commonly used to solubilize hydrophobic membrane proteins [8, 11]. Like lipids and membrane proteins, detergents are amphiphilic. They consist of a water-soluble head that contains polar, non-ionic, or ionic groups and a water-insoluble, non-polar tail. For the head groups, the following chemical motives are common: sulfates (anionic), sulfonates (anionic), carboxylates (anionic), quaternary amines (cationic), saccharides (non-ionic), amine oxids (zwitter-ionic), phosphocholine (zwitter-ionic), polyglycerols (non-ionic), polyethylenglycols (non-ionics) (Figs. 2–3). The non-polar tail often consists of a saturated linear alkyl chain or a cholesterol-based structural motive (Figs. 2–3). Following the motto *similia similibus solvuntur* (similar substances will dissolve similar substances), ionic detergents can solubilize protein-containing membranes. However, drawbacks can become apparent when the workflow moves on to proteolytic digest, LC separation, and MS analysis.

Drawbacks associated with the use of detergents are related to the properties of the detergent itself. High detergent concentrations can hinder protease activity which may limit protein digests required for BUP [8]. Furthermore, detergents can hamper MS analysis by inducing ion suppression and electrospray instability [8, 30]. The latter aspects are critical for BUP and TDP experiments but are principally relevant for all MS-based proteomics applications. From the perspective of detergent designers, these problems can be addressed in two ways: (i) chemically, for example, by modifying the molecular structure of detergents to make them more compatible to proteolytic digests and MS analysis. (ii) Practically, by optimizing the detergent handling, for example, by including detergent exchange or detergent removal steps prior to MS analysis. Recent MS detergent examples are discussed throughout the following sub-chapter.

### New detergents for BUP and TDP

The general utility of all detergent classes has been tested for BUP and TDP experiments, including anionic, cationic, zwitter-ionic, and non-ionic detergents [8]. Today, the most established detergent for BUP and TDP applications is anionic SDS. It efficiently denatures and solubilizes a multitude of proteins, including membrane proteins [8, 11]. However, it is not compatible to MS. Practically, the handling of SDS has been optimized to compromise its solubilizing properties and incompatibility to MS, for example, by lowering the concentration of SDS below 0.001% w/v prior to MS analysis or by applying detergent depletion strategies [31–33]. Among eight SDS depletion techniques, Kachuk et al. [33] identified acetone precipitation as an ideal strategy to increase the number of identifiable proteins and peptides in a proteomics workflow. Furthermore, SDS turned out to be compatible to suspension trapping (S-Trap)–based sample preparations and can improve BUP analysis [34]. Another method to remove SDS was developed by Kim et al. [35], where they coupled a membrane filter platform directly to the pump of their LC–MS device. Detergent depletion techniques can principally bias obtainable results, which is a motivation to chemically modify solubilizing detergents in such a way that they become MS-compatible upon degradation. Examples include acid-labile analogues of SDS, such as RapiGest, MaSdEs, and ProteaseMax (Fig. 2) [36–38]. These detergents are degraded post-trypsin digestion by acidifying followed by heating. Furthermore, these detergents show good solubilizing properties and can accelerate trypsin activity for the benefit of the digest [38, 39]. The ability to obtain both optimal protein solubilization and trypsin activity is depending on the detergent concentration and determined empirically [38–40]. A challenge commonly faced with acid-labile detergents is that the acidic conditions required to stop the digest may not be sufficient to completely degrade the detergent. The latter aspect can lead to unintended MS interference [39]. Furthermore, the ability to quantitively digest detergents depends not only on acidity but also on temperature and incubation time [36–38]. Incubation times can range from minutes to 24 hours [36–38]. Residues of degradable detergents may remain present in samples, including fragmentation products, and their effect on MS data quality cannot be generalized [39]. Alternatively, light has been established as a stimulus for detergent degradation. Brown et al. [41] designed an azo-containing analogue of SDS, namely Azo, with good solubilizing properties (Fig. 2). Upon irradiation with light, Azo degrades and the sample becomes MS-compatible, thus making it ideal for BUP and TDP experiments on membrane proteins [41, 42]. For example, Azo has enabled the comprehensive TDP analysis of ATP synthase subunit proteins from cardiac tissue and BUP analysis of membrane proteome from kidney cells [41, 42]. Recently Brown et al. [43] developed a non-ionic cleavable surfactant, containing a disulfide bond which can be cleaved by reduction prior to MS, reducing MS interference. Further studies will reveal the general utility of this approach for proteomics.

In clinical research, fresh frozen (FF) and formalin-fixed paraffin-embedded (FFPE) human tissues are stored for later analysis. Cross-linking and other reactions during sample preparation and storage hinder effective protein recovery. Dapic et al. [40] evaluated different acid-labile detergents and others in FF and FFPE human kidney tissue. Working on a similar issue, Liu et al. [44] recently presented a rapid digestion method, enabled by cationic C12Im-Cl (Fig. 2), which simplified the sample preparation of FFPE liver cancer tissues. Although the proteome coverage is slightly lower compared to the control, their findings pose a promising approach that enables the fast proteomics analysis of FFPE tissue within approximately 2 hours [44].

Alternative strategies for the optimization of MS detergents consider that every sample preparation strategy possesses an individual bias on obtainable results. Following this rationale, Choi et al. [45] suggest that a combination of structurally different detergents is required to match the structural diversity of the entire proteome for the benefit of the observable proteome. Alternatively, Khanal et al. [46] established fluoroalcohol-induced coacervation biphasic systems (FA*iC*-BPS) as a novel approach for BUP analysis. Instead of reconstructing the proteome by combining the proteomes observable from different detergents, the authors established a mixture of zwitter-ionic DMMAPS, quaternary ammonium salt (QUATS), and hexafluoroisopropanol (HFIP) to improve proteome coverage of yeast cells by 18% compared to a urea control and for the benefit of the observable membrane proteome[46].

Non-ionic detergents can be used for both, BUP and TDP [8]. Established non-ionic detergents include n-dodecyl-ß-D-maltoside (DDM), digitonin, Tween-20, PEG-4000, Brij-55, NP-7, and Triton X-100 [8, 10, 47–49]. For the analysis of the membrane-bound proteome, Pham et al. [47] outlined three characteristics that make Triton detergents useful for BUP and TDP analysis: (i) their wide use in membrane protein sample preparation, (ii) their phase separation properties which facilitate the enrichment of membrane proteins, and (iii) their non-ionic nature which makes them generally more MS-compatible than ionic detergents. Despite the latter beneficial characteristics, ionic detergents are currently the norm in BUP and TDP laboratories. To sum up, an optimal detergent for BUP and TDP solubilizes all proteins and does not interfere with MS measurements. The main applicatory difference of detergent between BUP and TDP applications is the digest. In the case of BUP applications, it is important to optimizing detergents for the benefit of protein solubilization, protease activity during the digest, and low MS interference. In the cases of TDP applications, it is more important to optimizing detergents for the benefit of protein solubilization while simultaneously lowering MS interference.

### Detergent requirements for nMS and Nativeomics

Unlike BUP and TDP, the concept of nMS and Nativeomics is to study intact, non-denatured membrane protein complexes (Fig. 1). Membrane proteins and their complexes are first purified in solution and then ionized and transferred into the vacuum of a mass spectrometer with the aim to maintain non-covalent interactions from solution species [50]. Since water-soluble proteins often ionize better than insoluble ones, membrane proteins are commonly overexpressed and purified from membranes with detergents to enable nMS and Nativeomics analysis[51]. The purified complexes can also be subjected to BUP and TDP analysis to validate protein identity and composition upon purification (Fig. 1). In addition, nMS and Nativeomics experiments can be used to confirm oligomeric states and to gain insights into identity and relative amounts of co-purified ligands. Once ligand identity is confirmed, biological relevance can be studied [14, 52–55]. In addition to detergents, the use of alternative membrane mimetics is gaining increasing attention in the field. For further insights into the utility of membrane mimetics for nMS and Nativeomics, we refer to the recent reviews on the topic [52–54, 56, 57].

Today, it is well established that the ability to study intact membrane protein complexes by nMS and Nativeomics depends not only on MS instrumentations but also on detergents [14, 55, 58]. Like for BUP and TDP, detergents need to compromise favorable solubilizing, denaturing, and MS-compatible properties. Unlike BUP and TDP, detergents need to preserve non-covalent protein–protein and protein–ligand interactions in solution and gas phase. Furthermore, in nMS and Nativeomics, detergents are removed inside the mass spectrometer by thermal activation and need to be compatible to the ionization technique, such as electrospray ionization (ESI). These requirements are commonly fulfilled by non-ionic detergents. Recent findings suggest that the use of submicron emitters can improve the applicability of ionic detergents for nMS of membrane proteins [59]. However, non-ionic detergents are still the norm for nMS and Nativeomics experiments, while ionic detergents are more common in BUP and TDP experiments (Fig. 1). Zwitter-ionic detergents represent a boarder case since they are formally charge-neutral but exhibit a stronger ionic character than non-ionic detergents. Some zwitter-ionic detergents have been established for BUP and TDP, such as ASB14 [45], DMMAPS [46], Zwittergent 3–10 [45], CHAPS [46], and PPS [40] (Fig. 2), while others are more common in nMS and Nativeomics, such as N-dodecyl-N,N-dimethylamine-N-oxide (LDAO) [60] and Fos-Choline (Fig. 3) [61].

Ideal detergents for nMS and Nativeomics enable the extraction and affinity purification of membrane proteins. Furthermore, they are compatible to ESI and can be readily removed from protein complexes inside the vacuum of a mass spectrometer. Like with other applications, the utility of detergents for membrane protein purification and nMS has initially been determined by empirical screening [13, 58, 60, 62]. To rationalize the role of the detergents, Reading et al. [60] found that the chemical nature of detergent head groups is a determining factor for the purification and nMS analysis of membrane proteins. Detergents commonly employed in purifications, for example, saccharide detergents, like n-dodecyl-ß-D-maltoside (DDM), n-dodecyl-ß-D-maltoside (DM), and lauryl maltose neopentyl glycol (LMNG), are not ideal for nMS [58, 60]. They require harsh activation conditions to be removed from protein complexes inside mass spectrometers, which can lead to unintended loss of non-covalent protein–protein and protein–ligand interactions in the gas phase [58, 60]. Other detergents, like tetraethylene glycol monooctyl ether (C8E4) and LDAO, require softer activation conditions compared to saccharide detergents and are more compatible to nMS [58, 60]. However, they tend to promote the loss of non-covalent protein–protein and protein–ligand interactions in solution. In summary, detergents that exhibit both favorable solution and gas-phase properties rarely exist [58]. From the perspective of detergent designers, these problems can be addressed in two ways: (i) chemically, for example, by modifying the molecular structure of detergents to outbalance optimal solution and gas-phase properties. (ii) Practically, by optimizing the detergent handling, for example, by using detergent mixtures or detergent exchange strategies. Recent MS detergent examples are discussed throughout the following sub-chapter.

### Designing detergents for nMS and Nativeomics

Keener et al. [57] recently summarized a general overview about common non-ionic and zwitter-ionic detergents and their utility for nMS of membrane proteins. Here, we complement this knowledge through a discussion of different perspectives that enable detergent designers to tune the performance of MS detergents for individual application in nMS and Nativeomics, including (i) delipidation, (ii) charge reduction, (iii) stickiness, (v) detergent exchange, and (vi) heterogeneity.

Stabilizing membrane proteins in solution during purification is an important requirement for MS detergents in nMS and Nativeomics. Membrane protein instability has been linked to the loss of protein-lipid interactions during purification [63, 64]. This suggests that detergents that efficiently co-purify lipids, such as DDM or LMNG, may provide better membrane protein stability than detergents that efficiently delipidate membrane proteins, such as n-octyl-β-D-glucoside (OG) and C8E4. Extensive lipid co-purification, however, can also hamper nMS experiments [65]. Lipids can strongly bind to membrane proteins in the gas phase. Consequently, the harshest possible activation conditions enabled by MS instruments may not be sufficient to obtain resolved spectra of heavily lipidated membrane proteins [58, 65]. This problem can be addressed in three ways: first, delipidation protocols with mildly delipidating detergents are applied until sufficiently resolved spectra are obtained [65]. Second, delipidation protocols based on the detergent exchange into strongly delipidating detergents are applied until sufficiently resolved spectra are obtained [66]. More quantitative detergent exchange is typically done by means of size-exclusion columns. More partial detergent exchange is done by means of Zeba Spin desalting columns or drop dilution [14, 67]. Third, a detergent is used that is designed to co-purify lipids and to be compatible to nMS so that sufficiently resolved spectra are obtained [51, 58, 68–70].

The first and second strategies require empirical testing of detergents and delipidation protocols. The third strategy requires newly designed detergents which have not yet been commercialized but can be synthesized by chemists [51, 58, 68–70]. Oligoglycerol detergents (OGDs) are one example of a newly designed detergent class for the purification and nMS analysis of membrane proteins. The key principle behind OGDs is that synthetic protocols enable chemists to readily change their molecular structure for structure–property studies [71]. The role of varying the OGD structure in protein experiments was understood retrospectively, i.e., after comparing protein purification and nMS analysis outcomes obtained from detergent screenings [58]. The outcome was condensed into design guidelines with which the structure of OGDs can now predictably be tuned for individual experimental parameters, including protein yields, delipidation, and charge reduction inside the vacuum of a mass spectrometer [58]. Conveniently, OGDs enable the straightforward nMS analysis of membrane proteins and their lipid complexes, which represents a new enabling step for the investigation of drug targets [27, 51, 58, 68–70, 72, 73].

Since delipidation can be a critical parameter, an alternative view on the optimization of detergents for membrane protein delipidation has recently been explored, which bases on the manipulation of the critical aggregation concentration (cac) [70]. The cac is the minimum concentration of a detergent required to form aggregates in solution. In this regard, the abbreviation for the critical micelle concentration (cmc) is more common in literature. The cmc is the minimum concentration of a detergent required to form micelles in solution. However, methods for the determination of the cac usually do not provide direct evidence for the aggregate morphology that is formed above the cac. However, since the minimum information required for the solubilization of membrane proteins is the cac of a detergent, regardless of the formed aggregate morphology, we preferred to use this abbreviation.

To prevent membrane protein precipitation in the absence of membranes, detergent concentrations in purification buffers are commonly kept above the cac [74]. At the beginning of a purification, higher detergent concentrations are used, typically in the amount of 1% w/v. During affinity purification, detergent concentrations are reduced to two times cac. Detergents compete with lipid molecules for binding to membrane proteins in a concentration-dependent manner [70]. Therefore, detergents with higher cac values, such as C8E4, [G1] OGD, OG, or LDAO, delipidate membrane proteins more efficiently than detergents with lower cac values, such as DDM, LMNG, and [G2] OGDs [70, 75]. This knowledge has recently been harnessed to tune the structure of OGDs and hybrid detergents for the benefit of reduced cac values and optimal delipidation for the nMS analysis of membrane protein-lipid complexes [58, 70]. Following this rationale, membrane proteins can also be delipidated with a mildly delipidating detergent by increasing its concentration in purification buffers [66] or by applying purification steps with the same detergent repetitively, such as in the case of multiple ultrafiltration steps [65, 76].

Furthermore, Urner et al. [69] found that the relative amount of co-purifying lipids is biased by the composition of the starting material used for purifications, i.e., protein-containing membrane suspensions. The authors found that *E. coli* membrane suspension contains small, water-soluble membrane structures and large, water-insoluble membrane structures. The co-solubilization of small and large membrane structures with detergents led to a substantial increase in the co-purification of membrane protein-lipid complexes compared to purifications in which membrane proteins were selectively solubilized from small, water-soluble membrane structures [69]. We expect this knowledge to be important for studies in which protein-lipid interactions detected by nMS and Nativeomics after purification from membrane suspensions with detergent are evaluated regarding their role in membrane protein structure and function.

Tuning delipidating to make co-purifying protein-lipid interactions analyzable by nMS and Nativeomics becomes increasingly important for integrated research approaches that aim for the investigation of complex membrane environments [52]: (1) the ability to design mildly delipidating detergents can provide insights into the lipid environment that co-purifies with membrane proteins. (2) The ability to design strongly delipidating detergents can be used to remove the lipidome surrounding membrane proteins, thus providing a source of lipid-free proteins for the reconstitution into in vitro membrane environments to unravel the roles of lipids in protein structure and function. In addition to delipidation, the stickiness of detergents in the gas phase of a mass spectrometer can be a determining factor for the ability the release membrane proteins from detergent micelles. It is generally accepted that electrostatic interactions between membrane proteins and detergents become significantly stronger during the transfer from solution into the vacuum of a mass spectrometer. Reading et al. [60] found that saccharide detergents require harsher activation conditions for detergent removal than LDAO or polyethylene glycol detergents. Urner et al. [77] confirmed that the energy required to break complexes formed between proteins and non-ionic detergents decreases when the number of hydroxyl groups in detergent head groups is reduced. Consequently, one design strategy for MS detergents for nMS and Nativomics is to reduce the stickiness by reducing the number of hydroxyl groups in detergent head groups. Detergent head groups with four hydroxyl groups or less, such as in the cases of C8E4, OG, or [G1] OGDs, are easier to remove from protein ions in the vacuum of mass spectrometer than detergents with seven hydroxyl groups or more, such as in the cases of DDM, LMNG, or [G2] OGDs (Fig. 3) [58, 60, 77]. In this regard, it is not only the number of hydroxyl groups in detergent head groups that matters. Also, the absolute concentration of detergents is important. For example, concentrating membrane proteins in detergent solutions in centrifugal filters frequently results in over-concentration of detergents even if the molecular weight cutoff of centrifugal filters is similar to or higher than the molecular mass of purified membrane protein-detergent complexes. Gobet et al. [78] recently outlined a solid framework to investigators to improve biochemical and structural studies of membrane proteins by considering the non-Newtonian behavior of detergents in context with the centrifugal force employed when working with centrifugal filters. We anticipate that avoiding detergent over-concentration can help to improve spectral quality in nMS and Nativeomics experiments and is particularly important when working with detergents that are referred to as sticky detergents, for example, [G2] OGDs, DDM, or LMNG.

A complementary design strategy for MS detergents is to employ functional groups that are capable of capturing charges in the gas phase, e.g., protons or sodium ions. For example, when ESI is operated in positive polarity mode (ESI +), charge-repulsive interactions between positively charged membrane protein ions and detergent ions lower the energy that is required to break protein-detergent complexes [77]. Tuning membrane protein charge states can be interesting for nMS and Nativeomics experiments for different reasons: first, charge-reducing detergents are often more MS-compatible than non-charge-reducing detergents due to charge-repulsive interactions that facilitate detergent removal inside the vacuum of a mass spectrometer [60, 77]. Second, charge reduction helps to prevent Coulomb-driven unfolding and dissociation processes, which can be a critical enabling step for the analysis of intact membrane protein complexes [79]. Third, charge reduction can increase the difference in the mass-to-charge ratio (*m/z*) between signals of membrane protein apo form, ligand-bound states, and post-translational modifications. The latter aspect can be important for the analysis of individual protein–ligand complexes or proteoforms whose *m/z* channels would overlap in the case of highly charged membrane protein ions [80]. Methods for membrane protein charge reduction include (i) increasing the basicity of the detergents’ functional groups to increase the affinity for capturing charge, for example, through the implementation of triazole, amine-oxide, or *cis*/*trans* azobenzene [58, 60, 68, 77], (ii) implementing charge-chelating groups into the detergent head group, such as in the case of C8E4 or OGDs, to increase the affinity for capturing charge [60, 77], (iii) treating the ESI plume with acetonitrile vapor [79], (iv) adding solution additives, such as imidazole and its derivatives [79, 81], amine oxides [82–84], amines [85, 86], and alkali metal acetate salts [87], and (v) detergent exchange from non-charge-reducing detergent into a charge-reducing detergent [60]. In addition, switching the ESI polarity can be an option to invert the charge-reducing properties of detergents [88]. Membrane protein charge reduction can be readily monitored by nMS when using the approaches described above. However, the underlying mechanisms are still under debate [61, 86] and it appears that the detergent tail has currently less relevance for MS compatibility compared to the detergent head group [61].

Since detergents that are suitable for nMS often provide more denaturing solution environments for membrane proteins than those that are suitable for purification, the detergent exchange can be beneficial to ensure the best detergent for an individual application is in place [66, 70]. For example, the detergent C8E4 provides a more denaturing solution environment for membrane proteins than the saccharide detergent DDM. However, DDM tends to be less suitable for the nMS analysis of membrane proteins. To compromise, membrane proteins can be purified by DDM followed by a detergent exchange into C8E4 to facilitate the nMS analysis. Detergent exchange strategies, including dialysis, size-exclusion chromatography, and drop dilution, usually do not enable a quantitative detergent exchange [89, 90]. After partial detergent exchange into a nMS-friendly detergent, membrane proteins can be sufficiently stable in solution over the time frame of a nMS experiment, i.e., minutes. In this way, the detergent exchange can be used to ensure the best detergent for an individual application is in place.

Finally, designing the heterogeneity of detergent batches that are used for the purification of membrane proteins and their analysis by nMS and Nativeomics did become more popular over the past years. Detergents are commonly designed to be homogenous. A traditional detergent batch contains one sort of molecule with defined head group and tail structure. This is the opposite of what lipid membranes surrounding membrane proteins are: heterogenous.

Attempts to increase detergent batch heterogeneity by mixing structurally different detergents turned out to be beneficial for the analysis of challenging membrane protein targets, such as G-protein-coupled receptors (GPCRs). Yen et al. [91–93] established the utility of a mixed micelle containing *n*-dodecyl-β-d-maltoside (DDM), cholesterol, and foscholine, for the purification and analysis of the human purinergetic receptor P2Y_1_ [91], the role of lipids in G-protein coupling and active states of GPCRs [92], and to decipher the biased and allosteric modulation of the ß_1_-adrenergic receptor in response to various ligands by nMS [93].

Urner et al. [58, 94] established a new synthesis strategy for the preparation of mixtures of OGDs regioisomers, which differ in terms of connectivity between glycerol units in their head groups (Fig. 3). OGD regioisomer mixtures turned out to be suitable for the extraction of large membrane protein quantities from biological membranes [58, 94]. More recently, Urner et al. [70] demonstrated that fusing different detergent head groups into hybrid detergents can serve as a new enabling step for the preparation of nMS-friendly detergents that can retain the beneficial properties of individual detergents and neglect some of their disadvantages (Fig. 3).

## Summary

**Fig. 4:**
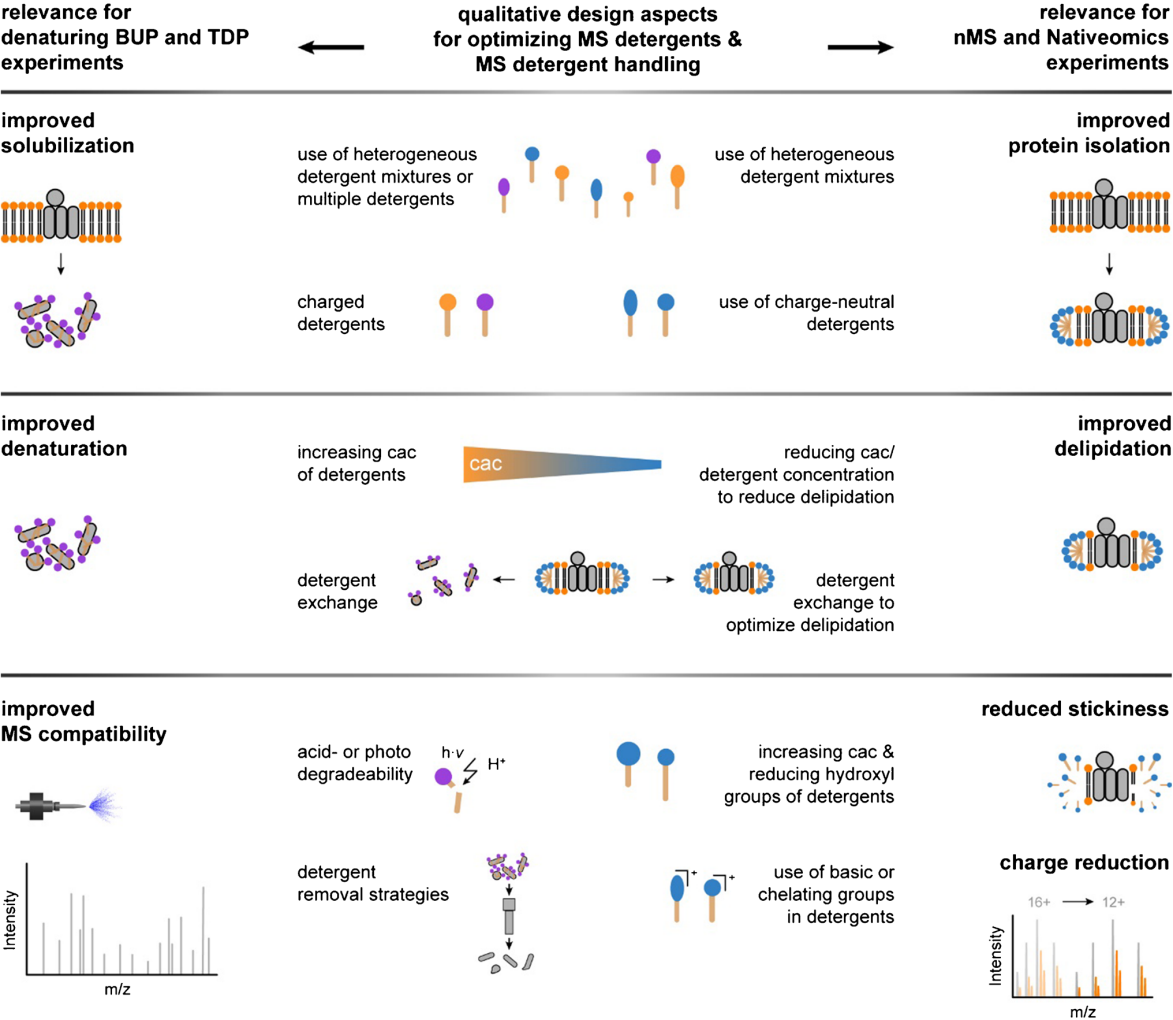
Rationalizing the design of mass spectrometry detergents. Schematic showing an overview about qualitative design aspects for MS detergents and their relevance for individual steps in BUP, TDP, nMS, and Nativeomics. The optimal design of MS detergents depends on the application, e.g., denaturing BUP and TDP (left) or nMS and Nativeomics (right)

In summary, a broad repertoire of design concepts for the optimization of MS detergents in BUP, TDP, nMS, and Nativeomics has been established (Fig. 4). Since the charge of the detergent head group is a determining factor for utility, MS detergents can be separated into two categories, e.g., those that are more suitable for denaturing BUP and TDP and those that are more suitable for nMS and Nativeomics (Fig. 1). Increasing the heterogeneity, charge, cac, degradability, and the implementation of detergent removal strategies are established design aspects for the optimization of MS detergents for individual steps in BUP and TDP experiments (Fig. 4). Varying the heterogeneity and cac of detergents, decreasing the charge and number of hydroxyl groups, and implementing basic or chelating groups are established design aspects for the optimization of MS detergents for individual steps in nMS and Nativeomics experiments (Fig. 4). We anticipate that the overview of qualitative design aspects provided by this review will support the optimization of MS detergents with tailor-made properties for the analysis of challenging biological systems (Fig. 4).

## Outlook

The development of design guidelines for the predictable optimization of detergent mixtures is still in its early stages. Recent case studies clarified that the use of heterogenous detergent batches can be more beneficial compared to the use of homogenous detergent batches. However, in the case of non-covalent detergent mixtures, optimal mixing ratios are determined empirically. Furthermore, in the case of covalent detergent mixtures, i.e., hybrid detergents, synthetic effort can be huge. Therefore, methodological improvements facilitating either the rationalization of the design in heterogenous detergent mixtures or the synthetic access to hybrid detergents will be key to unlock further advances. The ability to predictably tune detergent heterogeneity for the benefit of individual applications in BUP, TDP, nMS, and Nativeomics will serve as enabling step for future applications in biology and drug discovery research.
